# Work Stress as a Risk Factor for Cardiovascular Disease

**DOI:** 10.1007/s11886-015-0630-8

**Published:** 2015-08-04

**Authors:** Mika Kivimäki, Ichiro Kawachi

**Affiliations:** Department of Epidemiology and Public Health, University College London, 1-19 Torrington Place, London, WC1E 6BT UK; Department of Social and Behavioral Sciences, Harvard School of Public Health, Boston, MA USA

**Keywords:** Psychosocial stress, Occupational, Cohort study, Myocardial infarction, Stroke

## Abstract

The role of psychosocial work stress as a risk factor for chronic disease has been the subject of considerable debate. Many researchers argue in support of a causal connection while others remain skeptical and have argued that the effect on specific health conditions is either negligible or confounded. This review of evidence from over 600,000 men and women from 27 cohort studies in Europe, the USA and Japan suggests that work stressors, such as job strain and long working hours, are associated with a moderately elevated risk of incident coronary heart disease and stroke. The excess risk for exposed individuals is 10–40 % compared with those free of such stressors. Differences between men and women, younger versus older employees and workers from different socioeconomic backgrounds appear to be small, indicating that the association is robust. Meta-analyses of a wider range of health outcomes show additionally an association between work stress and type 2 diabetes, though not with common cancers or chronic obstructive pulmonary disease, suggesting outcome specificity. Few studies have addressed whether mitigation of work stressors would reduce the risk of cardiovascular disease. In view of the limited interventional evidence on benefits, harms and cost-effectiveness, definitive recommendations have not been made (e.g. by the US Preventive Services Taskforce) for the primary prevention of cardiovascular disease via workplace stress reduction. Nevertheless, governments are already launching healthy workplace campaigns, and preventing excessive work stress is a legal obligation in several countries. Promoting awareness of the link between stress and health among both employers and workers is an important component of workplace health promotion.

## Introduction

With most adults spending around half of their waking hours at work, the workplace is an important setting to promote health and well-being. Various national and international bodies are responsible for ensuring the health and safety of employees, with a focus on identifying physical, chemical and biological hazards in the workplace. Increasingly, attention is also being paid to the psychosocial work environment, with a major focus on work stress.

Research on stress and cardiovascular disease has a long history. Writing at the beginning of the twentieth century, Sir William Osler, the father of modern medicine, suggested that a major cause of myocardial infarction was the “wear and tear of life” [1]. More systematic research on work stress began in the late 1970s and early 1980s when Robert Karasek launched the job strain model [2, 3]. The model proposed that high psychological demands combined with low individual control over those demands leads to physiological strain, and hence, increased risk of cardiovascular disease. Subsequent research has broadened the concept of work stress beyond proximal job task characteristics to embrace organizational factors (such as work schedules) and even broader labour market arrangements (including job security and work-life balance) [4–6]. For example, the effort–reward imbalance model posits that experiencing an imbalance between high effort and low reward at work is stressful as it violates core expectations about reciprocity and adequate exchange at work [4]. Researchers have also examined job insecurity [5, 6] (which appears particularly relevant during economic downturns) and long working hours [7, 8] as risk factors for cardiovascular disease.

In this review, we summarize the quantitative evidence linking common work stressors to cardiovascular disease risk, with particular emphasis on estimates of effect size, consistency of findings, outcome specificity and underlying mechanisms. We also discuss potential weaknesses and gaps in studies in this field and consider implications for clinical practice and health policy. We close this review by outlining future directions for research on work stressors and cardiovascular health.

## Meta-analyses of the Association Between Work Stressors and Cardiovascular Disease

The strongest evidence for causation derives from experimental manipulation of an exposure (work stress) to see whether it can affect the outcomes of interest (e.g. cardiovascular disease incidence). Experimental evidence of this sort remains extremely sparse in the area of work stress [9]. Instead observational studies have provided the bulk of evidence on work stress and cardiovascular disease. We here limit our review to prospective cohort studies, as case-control and cross-sectional studies provide a more limited basis for causal inference.

Features that would support a cause and effect association between work stressors and cardiovascular disease include temporal sequence (the exposure precedes the onset of disease), consistency of results across different studies, biological plausibility, specificity (the association is observed in a specific set of diseases), reversibility (reduction of work stress reduces disease risk), and large effect sizes (typically a relative risk of >2 for exposed compared to unexposed groups) [10]. However, as Austin Bradford Hill was quick to caution, these are not “hard and fast” criteria but rather a heuristic set of guidelines for assessing causality. For example, the absence of specificity does not rule out a causal association, as some risk factors (e.g., cigarette smoking) are associated with multiple adverse effects.

We are not aware of large-scale randomized controlled trials on work stress and cardiovascular disease prevention, but some non-randomized natural experiments have been published. In a series of studies from Finland, the degree of organizational downsizing was used as proxy measures for work stress [11–14]. Analysis of 22,400 employees remaining in the organizations showed that cardiovascular mortality was 2.0 (95 % confidence interval 1.0 to 3.9) times higher after major downsizing than after no downsizing [14]. Splitting the follow-up period into two halves showed a 5.1 (1.4 to 19.3) times increase in cardiovascular mortality for major downsizing during the first 4 years after downsizing. No excess risk was observed during the second half of follow-up suggesting that major organizational downsizing may be a trigger of fatal cardiovascular events among vulnerable employees. However, these findings should be interpreted with caution as the number of cardiovascular deaths was small (*n* = 79), and thus, it is possible that the association was observed by chance.

**Fig. 1:**
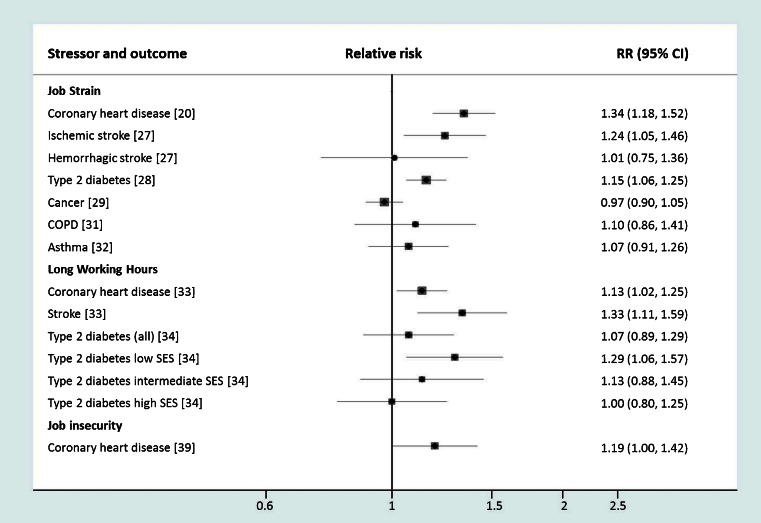
Associations of work stressors with cardiovascular disease and other chronic conditions in recent meta-analyses of prospective cohort studies. Reference in *parenthesis*

Numerous prospective cohort studies have examined the association between work stress and incident coronary heart disease (Fig. 1). The findings on job strain have been described in narrative reviews [15–17] and recently also in quantitative meta-analyses [18, 19••, 20]. The pooled relative risk of coronary heart disease across prospective studies is 1.34 (95 % confidence intervals 1.18–1.51) times higher for employees reporting job strain compared to those free of job strain [20]. In addition, an in-depth meta-analysis using individual participant data from almost 200,000 employees in eight European countries, the individual-participant data meta-analysis in working populations (IPD)-Work consortium, suggests that the association between job strain and incident coronary heart disease is similar in men and women, among younger and old, as well as across all levels of socioeconomic position [21••].

Relatively few studies have examined the association between job strain and stroke. An association between job strain or its components (ie, high job demands and low job control) and stroke has been observed only in some studies [22–26]. In the IPD-Work consortium analysis of 1.8 million person-years at risk (mean follow-up 9.2 years), 2023 first-time stroke events were observed [27]. Of them, 1049 were ischemic. The hazard ratio for job strain relative to no job strain was 1.24 (95 % confidence interval, 1.05 to 1.47) for ischemic stroke, but no statistically significant association was observed for hemorrhagic or overall stroke. Other meta-analyses provide further evidence of outcome specificity showing that job strain is associated with type 2 diabetes but not with common cancers, and the association with chronic obstructive pulmonary disease or inflammatory diseases is not statistically significant [28–32].

Several recent studies have examined the association between long working hours (typically referring to over 48 or 55 working hours per week) and risk of cardiovascular diseases. An early meta-analysis of 4 prospective studies noted a relative risk of 1.39 (95 % CI 1.12 to 1.72) for individuals working long hours compared to those working standard hours while the corresponding relative risk in the 7 case–control studies was higher, 2.43 (95 % CI 1.81 to 3.26) [7]. A more recent meta-analysis from the IPD-Work consortium was based on 22 prospective cohort studies with a total of 600,000 men and women from Europe, the USA and Australia. The summary relative risk for working long hours (55 h per week or more) compared to standard 35–40 h was 1.13 (95 % confidence interval 1.02 to 1.26), although a stronger association was observed among low socioeconomic status workers [33]. In contrast, long working hours were robustly associated with increased risk of stroke across all socioeconomic groups, men and women, as well as younger and older employees [33]. The overall relative risk of stroke associated with long working hours was 1.33 (95 % CI 1.11 to 1.61). The association between long working hours and type 2 diabetes was apparent only in individuals in the low socioeconomic status groups [34].

Prior to 2013, few published studies had examined the relation between job insecurity and coronary heart disease [22, 35–38] and of these, the two largest studies found job insecurity to be associated with higher, albeit statistically non-significant, risk of incident coronary heart disease [22, 36]. Subsequently, a meta-analysis of all prospective studies published and unpublished data from the IPD-Work consortium was based on 17 cohort studies and suggested that the relative risk associated with job insecurity is 1.19 (95 % CI 1.00 to 1.42) [39]. The association between job insecurity and coronary heart disease was partly explained by poorer socioeconomic circumstances and less favourable risk factor profiles among people with job insecurity.

Taken together, the excess cardiovascular disease risk for individuals exposed to work stressors—including job strain, long working hours or job insecurity—seems to be in the ball park of around 10–40 % compared with those free of such stress. This association is observed for a broad cross-section of workers, i.e. equally in men and women, young and old and across the socioeconomic spectrum. Meta-analyses of a wider range of health outcomes indicate specificity of the association for cardiometabolic diseases. The I^2^ statistics indicate that heterogeneity in effect estimates between studies is small suggesting that the evidence is consistent or reasonably consistent [20, 33, 39].

## Plausible Biological Mechanisms

Atherosclerosis, including the thickening of arterial walls and development of plaques, is a common pathology in cardiovascular disease. Measurement of carotid intima media thickness (IMT) is feasible in cohort studies because it can be carried out noninvasively using external ultrasound. In an investigation of young adults, job strain was cross-sectionally associated with increased IMT in men but not women [40]. However, other investigations focusing on middle-aged adults found no consistent association between workplace or perceived stress and carotid IMT in men or in women [41, 42].

There is some evidence of a link between stress and the metabolic syndrome, which is commonly defined as having at least three risk factors among central obesity, hypertension, hyperglycemia, elevated triglycerides and low levels of high-density lipoprotein (HDL) cholesterol. In the Whitehall II study of British civil servants, work stress defined by job strain combined with low social support (called iso-strain) was associated with the onset of the metabolic syndrome in a dose–response manner: the more frequently a participant reported stress over repeated waves, the higher was the risk of metabolic syndrome by the end of follow-up [43]. In the same study, metabolic syndrome explained approximately 15 % of the association between iso-strain and coronary heart disease [44•]. Iso-strain was also associated with the components of metabolic syndrome, such as higher waist (central obesity), high body mass index (general obesity) and dyslipidemia [44•]. However, a recent systematic review and meta-analysis of cohort studies on job strain failed to confirm a longitudinal association with obesity or weight gain in the totality of studies including over 60,000 men and women [45]. Similarly, a consortium study of 150,000 men and women from high, middle and low-income countries concluded that individuals with permanent life stress were slightly more obese, but there was no overall independent effect and no evidence that abdominal obesity would increase with higher levels of stress [46]. This was also the case for life and work stressors in a meta-analysis of published studies [47]. In the IPD-Work consortium, job strain was cross-sectionally associated with both obesity and underweight, suggesting that stress might be related to weight gain in some individuals but weight loss in others. Because all the observed associations were relatively weak, weight change alone might account only for a negligible part of the excess coronary heart disease risk among individuals with job strain.

Studies measuring blood pressure during the working day suggest that work stress is associated with small increases in blood pressure, but that these do not necessarily translate into clinical hypertension [48•, 49]. In the IPD-Work consortium, for example, people with job strain were more likely to have diabetes (odds ratio 1.29; 95 % CI 1.11 to 1.51), to smoke (1.14; 1.08 to 1.20), be physically inactive (1.34; 1.26 to 1.41) and to have an elevated Framingham risk score (1.13; 1.03 to 1.25), but no robust association with hypertension was observed [50]. In relation to other work stressors, such as job insecurity, stressed participants were less likely to be physically active and have a higher prevalence of hypercholesterolaemia and hypertension compared to individuals who are free of work stress [39]. However, there was no longitudinal data to confirm the temporality between job insecurity and these risk factors.

In sum, no single biological mechanism has been found to link work stressors to cardiovascular diseases. Rather there may be multiple stress-related pathways which can act as contributing factors affecting the aetiology but also triggering cardiovascular events among vulnerable individuals [19••, 51]. In addition, work stress may induce biological changes indirectly via affecting lifestyle factors. For example, employees experiencing job strain may be more likely to become physically inactive, compared to those free of job strain [52], and there are also modest associations of work stressors with higher smoking intensity and higher alcohol intake [8, 53, 54]. Further research is needed to understand the biological mechanisms linking chronic stress to disease outcomes (which may differ from acute stress mechanisms that are typically examined in the laboratory setting).

## Assessment of Bias and Confounding

Bias and confounding are important considerations when evaluating observational epidemiological evidence. For example, Ioannidis has provocatively argued that most observational research findings are false and that bias and confounding are more likely when the effect sizes are small, there are a greater number of associations to be tested and there is more flexibility in defining exposures or specifying analytical models [55•]. These issues might also affect the evidence on work stressors and cardiovascular disease. For example, there is no consensus on how best to operationalize job strain. Fransson et al. identified 11 different sets of job strain items [56] and even when the item content is identical, at least four alternative ways of defining job strain have been used [57]. This is problematic as alternative versions can open the door for the use of post hoc comparisons and selective reporting of findings after multiple testing, thereby increasing the likelihood of false positive findings. Similarly, there can be citation bias in the literature regardless of the scientific quality of studies. A bibliometric analysis of prospective cohort studies on job strain and incident coronary heart disease showed that higher-quality science in this field did not garner more citations [58]. In contrast, studies that reported higher risk estimates were cited more frequently than those that reported lower risk estimates.

The IPD-Work consortium is an attempt to minimize biases and confounding that often affect observational studies. The hypothesized link between job strain and coronary heart disease was tested by extracting data from participating cohort studies in two stages: first, the exposure was harmonized across cohorts in a validation study, with investigators masked to outcome information; then, the endpoint of coronary heart disease was harmonized. To evaluate publication bias, both published and unpublished data were used. To reduce random error and allow subgroup analyses, the largest available databases to date were used (197 000 study members contributing 2350 events). To reduce the possibility of reverse causation (i.e. biases arising from the effect of undiagnosed coronary heart disease on perception of job strain), disease events that occurred in the first years of follow-up (left-censoring) were excluded from the analyses.

**Fig. 2:**
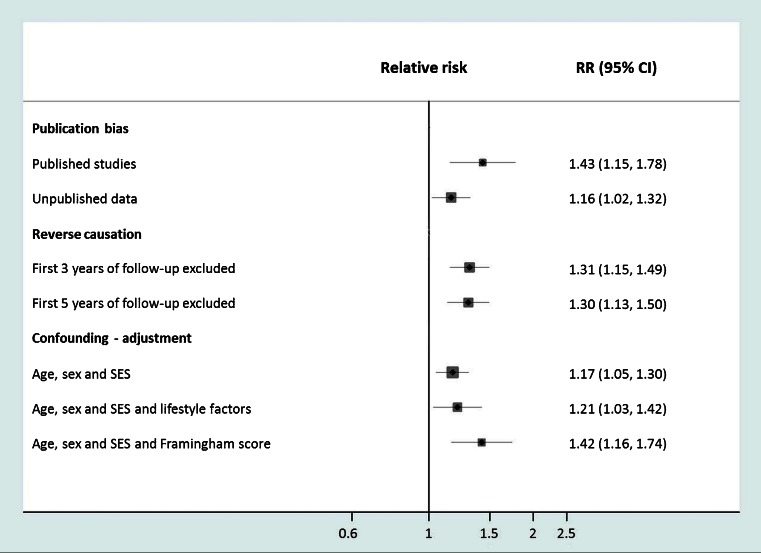
Association of job strain with incident coronary heart disease risk in relation publication status for data, reverse causation testing and adjustments. (Adapted from: Kivimäki M, Nyberg ST, Batty GD, et al. Job strain as a risk factor for coronary heart disease: a collaborative meta-analysis of individual participant data. Lancet. 2012; 380: 1491–7) [21••]

As shown in Fig. 2, there was some suggestion of publication bias. In three studies included in IPD-Work that had been published previously, the relative risk for coronary heart disease in those reporting job strain compared to those who did not was 1.43 (95 % CI 1.15 to 1.77). The hazard ratio based on ten studies that had not been published previously was smaller although still statistically significant, 1.16 (95 % CI 1.02 to 1.32). The combined relative risk was 1.23 (95 % CI 1.10 to 1.37). Excluding the first 3 or 5 years of follow-up had little effect on the association suggesting that reverse causation is an unlikely explanation for the link between job strain and coronary heart disease. Similarly, adjustment for baseline socioeconomic and lifestyle factors or standard risk factors indicated by the Framingham risk score did not attenuate the relative risk and thus the association seemed not to be confounded.

In conclusion, there is no strong evidence to suggest that the summary estimates for the association between work stressors and cardiovascular disease would be importantly confounded or biased. In view of the large sample size in the pooled evidence, it is also unlikely that the association would have been observed by chance.

## Implications for Clinical Practice, Policy and Future Research

Regardless of where one stands on the evidence linking work stress to cardiovascular disease, the mitigation of work stress would seem to be a desirable objective for the purpose of promoting the quality of life of workers worldwide. Indeed in many countries, preventing excessive work stress is a legal obligation. The European Union Working Time Directive also provides employees the right to limit their average weekly working time to 48 h, and the European Agency for Safety and Health at Work have launched the healthy workplaces campaign 2014–2015 to promote psychosocial work environment.

However, it is a separate question whether work stress also ought to be considered as a major target for cardiovascular disease prevention. Prevention strategies are based on an evaluation of the evidence on benefits, harms and cost-effectiveness using systematic and transparent approaches, such as the grades of recommendation assessment, development and evaluation (GRADE) system [59]. Evaluation for scientific quality begins with a systematic review of the best available evidence for a given risk factor. In GRADE, this evidence is initially graded on the basis of the strength of the study design with randomized trials representing “high quality” and observational studies denoted as being “low quality”. The initial grade is downgraded if there are serious limitations in the evidence, but there are also issues that can lead the quality grade to be upgraded.

According to GRADE, a major limitation of research on the effects of work stressors on coronary heart disease and stroke is the lack of randomized controlled trials. This has limited our ability to confirm the causal nature of the association between work stressors and cardiovascular disease, and to evaluate the extent to which interventions to reduce work stress would reduce disease risk. Without evidence from intervention studies, the GRADE conclusion that applies to the evidence to date is that “the true effect is likely to be close to the estimate of the effect, but there is a possibility that it is substantially different” (p404) [59]. Although there is no definitive indication for stress reduction in the primary prevention of cardiovascular events in clinical practice, promoting awareness of the link between stress and health would seem to be a worthwhile goal in workplace wellness promotion.

**Fig. 3:**
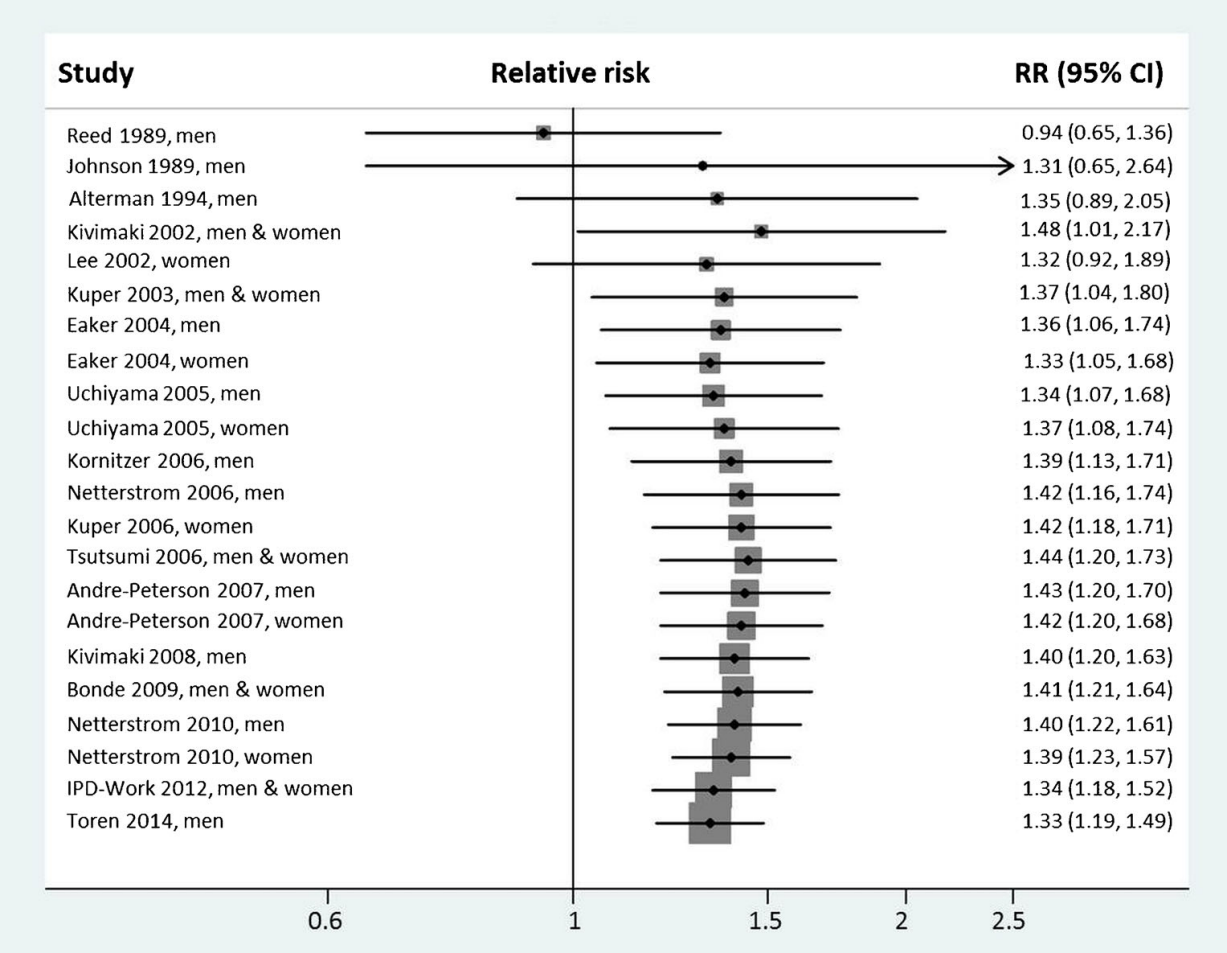
Cumulative meta-analysis of cohort studies on job strain and incident coronary heart disease. Full references for the constituent studies are available in Appendix 1

What are the next steps in research on work stress? Our cumulative meta-analysis of job strain and coronary heart disease suggests that little new will be gleaned by conducting additional studies performed in a similar way (Fig. 3). This is because the effect estimate in the cumulative evidence has been unchanged since 2006 after inclusion of the last 21 studies. In line with this, recent developments building upon the framework of the job strain model include proposals of broadening the measurement to additional features of work organization. We encourage investigators in the field to focus on potential psychological, behavioural and biological mediating mechanisms linking work stress to health outcomes; this is a major gap in the current evidence base. We also encourage stress researchers to conduct intervention studies to determine whether the observed associations can be replicated in experimental designs, such as individual RCT, cluster-randomized trials or natural experiments. Such studies come with great challenges, but they are needed to advance research in a field which hitherto has been dominated by observational evidence.

## Conclusions

The role of work stressors in generating adverse chronic health conditions has been subject to considerable debate. Many researchers argue in support of a causal connection while others remain skeptical and have argued that the effect on specific health conditions is either negligible or confounded. This review of evidence from Europe, the USA and Japan suggests that work stressors, such as job strain and long working hours, are associated with a moderately elevated risk of incident coronary heart disease and stroke. Differences between men and women, younger versus older employees and workers from different socioeconomic backgrounds appear to be small, suggesting that the association is robust. Furthermore, heterogeneity in effect estimates between studies is small suggesting that the evidence is consistent or reasonably consistent. In view of the limited interventional evidence on benefits, harms and cost-effectiveness, definitive recommendations have not been made (e.g. by the US Preventive Services Taskforce) for the primary prevention of cardiovascular disease via workplace stress reduction. However, the obligation of minimizing excessive stress at workplaces is a moral principle which is not dependent on the effects of work stress on cardiovascular health.

## Appendix 1. Studies Included in the Cumulative Meta-analysis Described in Fig. 3

Reed DM, LaCroix AZ, Karasek RA, Miller D, MacLean CA. 1989. Occupational strain and the incidence of coronary heart disease. Am. J. Epidemiol. 129:495–502

Johnson JV, Hall EM. 1988. Job strain, work place social support, and cardiovascular disease: a cross-sectional study of a random sample of the Swedish working population. Am. J. Public Health 78:1336–42

Alterman T, Shekelle RB, Vernon SW, Burau KD. 1994. Decision latitude, psychologic demand, job strain, and coronary heart disease in the Western Electric Study. Am. J. Epidemiol. 139:620–7

Kivimäki M, Leino-Arjas P, Luukkonen R, Riihimäki H, Vahtera J, Kirjonen J. 2002. Work stress and risk of cardiovascular mortality: prospective cohort study of industrial employees. Brit. Med. J. 325:857

Lee S, Colditz G, Berkman L, Kawachi I. 2002. A prospective study of job strain and coronary heart disease in US women. Int. J. Epidemiol. 31:1147–53

Kuper H, Marmot M. 2003. Job strain, job demands, decision latitude, and risk of coronary heart disease within the Whitehall II study. J. Epidemiol. Commun. Health 57:147–53

Eaker ED, Sullivan LM, Kelly-Hayes M, D’Agostino RB, Sr., Benjamin EJ. 2004. Does job strain increase the risk for coronary heart disease or death in men and women? The Framingham Offspring Study. Am. J. Epidemiol. 159:950–8

Uchiyama S, Kurasawa T, Sekizawa T, Nakatsuka H. 2005. Job strain and risk of cardiovascular events in treated hypertensive Japanese workers: hypertension follow-up group study. J. Occup. Health 47:102–11

Kornitzer M, deSmet P, Sans S, Dramaix M, Boulenguez C, DeBacker G, et al. 2006. Job stress and major coronary events: results from the Job Stress, Absenteeism and Coronary Heart Disease in Europe study. Eur. J. Cardiovasc. Prev. Rehabil. 13:695–704

Netterstrom B, Kristensen TS, Sjol A. 2006. Psychological job demands increase the risk of ischaemic heart disease: a 14-year cohort study of employed Danish men. Eur. J. Cardiovasc. Prev. Rehabil. 13:414–20

Kuper H, Adami HO, Theorell T, Weiderpass E. 2006. Psychosocial determinants of coronary heart disease in middle-aged women: a prospective study in Sweden. Am. J. Epidemiol. 164:349–57

Tsutsumi A, Kayaba K, Hirokawa K, Ishikawa S. 2006. Psychosocial job characteristics and risk of mortality in a Japanese community-based working population: the Jichi Medical School Cohort Study. Soc. Sci. Med. 63:1276–88

Andre-Petersson L, Engstrom G, Hedblad B, Janzon L, Rosvall M. 2007. Social support at work and the risk of myocardial infarction and stroke in women and men. Soc. Sci. Med. 64:830–41

Kivimäki M, Theorell T, Westerlund H, Vahtera J, Alfredsson L. 2008. Job strain and ischaemic disease: does the inclusion of older employees in the cohort dilute the association? The WOLF Stockholm Study. J. Epidemiol. Commun. Health 62:372–4

Bonde JP, Munch-Hansen T, Agerbo E, Suadicani P, Wieclaw J, Westergaard-Nielsen N. 2009. Job strain and ischemic heart disease: a prospective study using a new approach for exposure assessment. J. Occup. Environ. Med. 51:732–8

Netterstrom B, Kristensen TS, Jensen G, Schnor P. 2010. Is the demand-control model still a useful tool to assess work-related psychosocial risk for ischemic heart disease? Results from 14 year follow-up in the Copenhagen City Heart study. Int. J. Occup. Med. Environ. Health 23:217–24.

IPD-Work Consortium (10 unpublished studies, previously published studies excluded): Kivimäki M, Nyberg ST, Batty GD, Fransson EI, Heikkila K, Alfredsson L, et al. 2012. Job strain as a risk factor for coronary heart disease: a collaborative meta-analysis of individual participant data. Lancet. 380:1491–7.

Torén K, Schiöler L, Giang WK, Novak M, Söderberg M, Rosengren A. 2014. A longitudinal general population-based study of job strain and risk for coronary heart disease and stroke in Swedish men. BMJ Open.;4:e004355.
